# Supplementary figures and images for: Role of HO-1 against Saturated Fatty Acid-Induced Oxidative Stress in Hepatocytes
Source: Nutrients. 2021 Mar 19;13(3):993. doi: 10.3390/nu13030993 (PMC8003531; doi:10.3390/nu13030993)

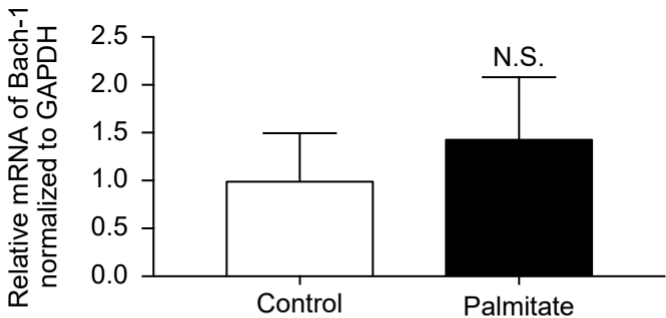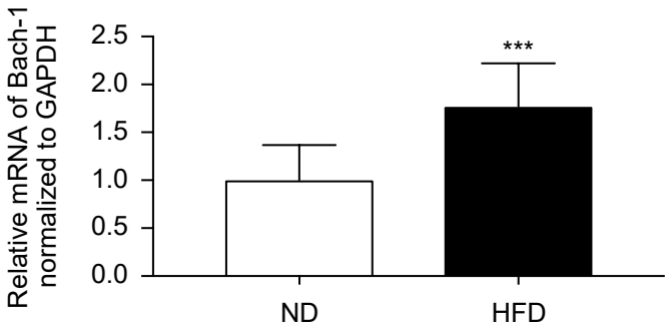

Supplement: Supplementary file 1 [file nutrients-13-00993-s001.zip › Supp figure 1,2/Supp Figure 2.pdf]

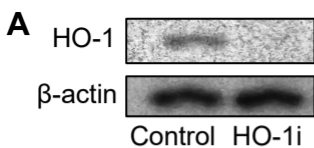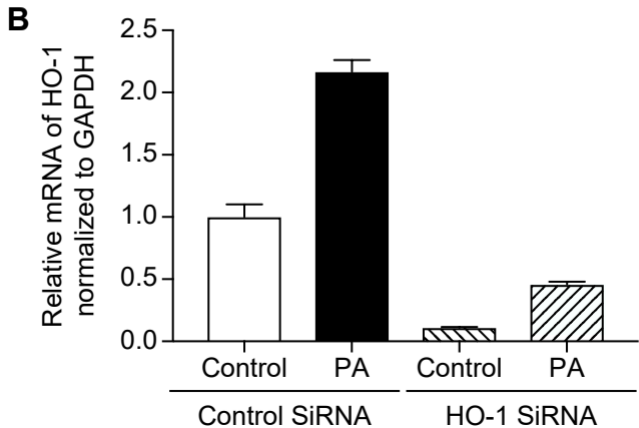

Supplement: Supplementary file 1 [file nutrients-13-00993-s001.zip › Supp figure 1,2/Supp Figure 1.pdf]
